# The Indications for Trional

**Published:** 1893-12

**Authors:** 


The Indications for Trional.—The recent tests of trional point to its special value in moderate or severe conditions of melancholic or hypochondriacal depression, in which case Brie and others state that it is always successful in doses of one to two grams. Sometimes a dose of two grams, given in the beginning, controls the condition so well that the following doses need not exceed one gram. That group of patients usually spoken of as having "melancholia with agitation" are also especially amenable to the trional treatment, even in cases which had proved refractory to treatment by opium, morphine, paraldehyde, and amyl hydrate. In maniacal excitement, inclusive of paralytic mania, doses of two and even three grams were sometimes required, especially when the restlessness was very marked. In hallucinatory dementia and paranoia, from one to three grams of trional gave satisfactory results. It is indicated in simple agrypnia (one to two gram doses), and in insomnia, with restlessness and excitement, as often observed in persons suffering from organic disorders. Trional causes no nausea, vertigo, or gastric disturbance of any kind, and, in suitable doses, causes no more than a transient drowsiness, which passes off after eating.—*Ex.*

